# Getting closer to the goal by being less capable

**DOI:** 10.1126/sciadv.aau5902

**Published:** 2019-02-06

**Authors:** Pedro D. Manrique, Mason Klein, Yao Sheng Li, Chen Xu, Pak Ming Hui, Neil F. Johnson

**Affiliations:** 1 Physics Department, University of Miami, Coral Gables, FL 33126, USA.; 2 College of Physics, Optoelectronics and Energy, Soochow University, Suzhou 215006, China.; 3 Department of Physics, The Chinese University of Hong Kong, Shatin, Hong Kong SAR, China.; 4 Physics Department, George Washington University, Washington D.C., 20052, USA.

## Abstract

Understanding how systems with many semi-autonomous parts reach a desired target is a key question in biology (e.g., *Drosophila* larvae seeking food), engineering (e.g., driverless navigation), medicine (e.g., reliable movement for brain-damaged individuals), and socioeconomics (e.g., bottom-up goal-driven human organizations). Centralized systems perform better with better components. Here, we show, by contrast, that a decentralized entity is more efficient at reaching a target when its components are less capable. Our findings reproduce experimental results for a living organism, predict that autonomous vehicles may perform better with simpler components, offer a fresh explanation for why biological evolution jumped from decentralized to centralized design, suggest how efficient movement might be achieved despite damaged centralized function, and provide a formula predicting the optimum capability of a system’s components so that it comes as close as possible to its target or goal.

## Teaser

A decentralized system of heterogeneous components is more efficient when they are less sophisticated.

## INTRODUCTION

Tragic events, such as the killing of a pedestrian by a driverless Uber in Tempe, Arizona on 18 March 2018, can shake public and political enthusiasm for driverless vehicles and planes (*1*). The need for accurate, safe, and efficient navigation is the key hurdle for these systems (*2*–*11*). Centralization—for example, by aggregating the local information processing of a system’s *N* ≫ 1 components into a centralized unit such as a brain or a central processing unit, by aggregating the local sensing of *N* ≫ 1 autonomous sensors into a centralized detector such as an eye, or by aggregating the local action of *N* ≫ 1 autonomous actuators into a centralized machine such as a leg—has many advantages. For example, a human relies on a centralized processor (brain), sensors (eyes), and actuators (legs) to walk in a straight line from A to B.

However, centralization has the disadvantage of making an entity vulnerable to damage or targeted attack—for example, an external hack that shuts down a cyberphysical system, a brain disease that leaves someone unable to move properly, eye loss that leaves them blind, the inevitable crash of a conventional car or plane following loss of its pilots or drivers, or the crippling of major areas of U.S. socioeconomic life when there is a federal shutdown. Centralized structures can also be disadvantageous in terms of the cost (e.g., energy or money) needed to maintain them, the need for localized heat dissipation, or the substantial communications bandwidth required to continually transport information into and out from the central unit (*12*). Delays in this information transfer can even produce dangerous system-level behavior (*5*, *9*).

## DECENTRALIZED NAVIGATIONAL MODEL

**Fig. 1:**
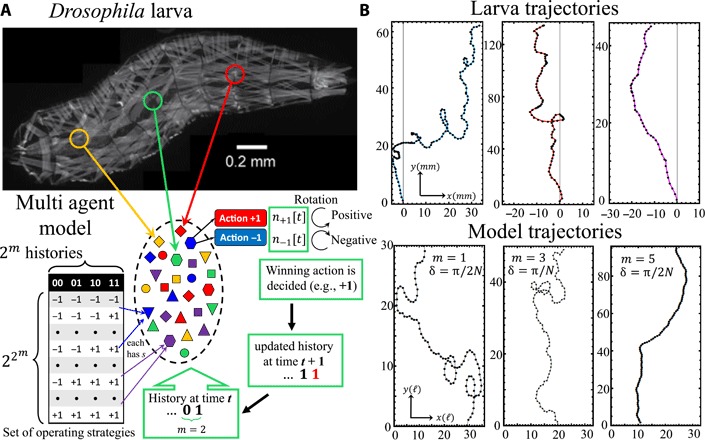
Modeling a segmented larva organism as a collection of heterogeneous semi-autonomous agents. (**A**) Top: Segmentation image from fluorescence micrograph of *w;Mhc-GFP*^*c*110^*/CyO* larva [adapted from (*23*)]. Bottom: Schematics of our multiagent, decentralized model that determines the system’s direction of motion toward a desired target. Heterogeneity of the agents mimics heterogeneity in regions of larva body wall that contribute to crawling or heterogeneity of member components (e.g., paddlers) in some locomotive device (e.g., canoe) or, more abstractly, members of some human group or organization lacking top-down control. Each agent acts as a limited sensor that captures the current bit of common information, as a limited information processor by having a memory that stores the most recent *m* bits of common information (i.e., history) and processes which action (±1) to take based on its best-performing algorithm (strategy) among the *s* that it has and as a limited actuator by trying at each time step to rotate the system an angle δ clockwise (action −1) or counterclockwise (action +1). Which action it takes depends on the current history at that time step (i.e., the common bit-string of length *m*) and its *s* algorithms (i.e., strategies), which form a look-up table for the action. *s* strategies are assigned randomly to each agent at the beginning, hence introducing intrinsic agent heterogeneity. (**B**) Top: Positive thermotaxis trajectories of *Drosophila* larvae. Data points are spaced in time by intervals of 5 s. Bottom: Model trajectories for different values of the agent capability *m* and different δ. *s* = 2, *N* = 101, and *q* = 0.3 (see text). Distance units are measured in model steps *ℓ*.

We consider a generic decentralized entity (Fig. 1A, bottom) inspired by the *Drosophila* larva (*12*–*26*) (Fig. 1A, top), which is able to execute exploratory routines (*20*) as a result of the aggregate actions of abdominal and thoracic network segments, that balances the tasks of runs and turns, acting like autonomous agents (i.e., klinotaxis) (*21*–*28*). Within the framework of biologically inspired algorithms, our approach lies within the category of foraging algorithms originally inspired by the chemotactic behavior (i.e., odor-driven foraging associated to food, habitat, and survival) exhibited by decentralized bacterial colonies such as *Escherichia* *coli* (*29*). In an engineering context of navigational algorithms, our model follows the principle of proportional navigation, which aims at preserving and maximizing a vehicle-target alignment (*30*). Our goal is not to provide an anatomically or physiologically precise description but rather to study the dynamics of a minimal, generic abstraction. The entity comprises an arbitrary number *N* of individual components or members, which we refer to as agents, since each has a limited ability to receive information from the outside (i.e., sensor), to store and process this information, and to act on this (i.e., actuator) by trying to push the system to the left or right.

**Fig. 2:**
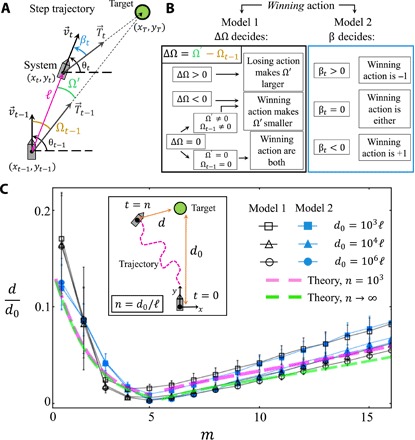
Details of our decentralized model and performance. (**A**) Key quantities in our model for a decentralized entity moving toward a target. (**B**) Rules for determining the winning action for model version 1 and model version 2. (**C**) Average ratio of the final to initial system-target distance (*d*/*d*_0_; see inset) as a function of agent capability parameter (i.e., memory) *m*. Averages are calculated over different initial conditions for model version 1 (black symbols) and model version 2 (blue symbols). *N* = 101, *s* = 2, and δ = π/2*N*. The system is initially positioned at the origin of the coordinate plane with a direction θ_0_ = π/2, which points toward the target sitting at (*x*_*T*_ = 0, *y*_*T*_ = *d*_0_). Averages are calculated over 100 initial strategy distributions and 100 initial winning histories for several values of *m* and *d*_0_. Pink and green dashed curves are results from our crowd-anticrowd theory for *n* = 10^3^ and up to second order in correlations and the limiting case for *n* → ∞, respectively.

Each agent’s capability is characterized by the integer *m*, since each agent can store the history of the past *m* outcomes for the entity, with each outcome being 0 or 1. The ability for even simple materials to have such a memory is well known, e.g., shape memory materials (*31*, *32*). Following the aggregate action of all the *N* agents (Fig. 2), the outcome is 1 if the entity becomes more aligned with the target and 0 if it becomes less aligned. There are hence 2^*m*^ possible histories of the past *m* outcomes, e.g., for *m* = 2, these are 00, 01, 10, and 11 (*33*, *34*). Each agent stores the same history at any given time step (e.g., 01) but has two possible actions that it can then take: to try to rotate the system an angle δ clockwise (action −1) or counterclockwise (action +1). Hence, there is a look-up table (Fig. 1B) in which each row is effectively an information-processing algorithm (referred to as a “strategy”) for predicting the next best action given one of the 2^*m*^ possible history inputs, meaning that there are $2^{2^{m}}$ possible strategies. Each agent is initially assigned *s* strategies randomly from the space of $2^{2^{m}}$ possible strategies, thereby introducing a heterogeneity in agents’ actions for the same history bit-string, which is again a known feature for smart materials. By allowing each agent to use the best performing among its *s* > 1 strategies at any given time step, and scoring all strategies based on their previous predicted actions, each agent has some adaptability. The agents’ heterogeneity is also consistent with *Drosophila* larvae in that different individual segments contract or extend on either side to adjust the orientation (Fig. 1A) and thus generate motion to the left or right—and also, in a more abstract sense, the different pieces of the neuronal network. Different neurons in *Drosophila* can be functionally different (*13*–*15*, *20*). At each time step, the system rotates under the aggregate action of the *N* agents and moves in a straight distance *ℓ* in the new direction. This space need not be geographical: It could be an abstract space spanned by performance variables in the more abstract setting of an entity of *N* individual humans in an organization or company with no top-down control, aiming for some particular collective target. Denoting the number of agents taking action ± 1 at time step *t* as *n*_± 1_[*t*], the entity’s overall rotation is then (*n*_+ 1_[*t*] − *n*_− 1_[*t*])δ. If all agents take the same action, for δ = π/*N*, then the system rotates 180°. The winning action is the one that improves the system-target alignment (see Fig. 2).

Figure 1B demonstrates the similarity between the trajectories generated by our generic model (bottom) and those of living *Drosophila* larva in our experiments (top). The larvae naturally seek out temperatures that maximize their growth rate (≈ 24*°*C) (*23*). They crawl atop a 22 cm by 22 cm agar substrate while experiencing a one-dimensional thermal gradient ranging from 17.5° to 22°C (i.e., positive thermotaxis) (*16*, *17*, *26*). The trajectories are captured by high–pixel density video cameras taking 15 frames per second. By choosing the target to be far away (0, 10^4^*ℓ*), the model mimics the one-dimensional gradient of the experiment.

**Fig. 3:**
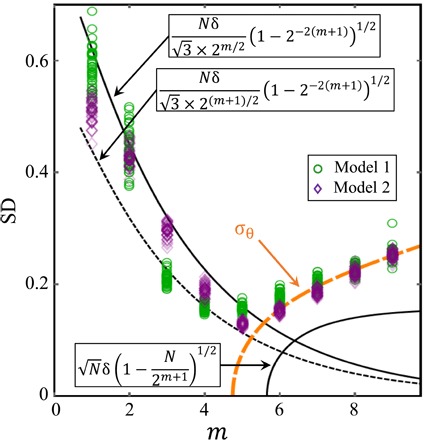
Dependence of the fluctuations in the system’s direction of motion (σ_θ_) with the agent capability parameter *m* using simulations (colored symbols) and our analytic crowd-anticrowd theory σ_*CA*_ (black lines). For larger values of *m*, additional temporal correlations appear: Adding these into the crowd-anticrowd theory improves the fit (orange curve).

**Fig. 4:**
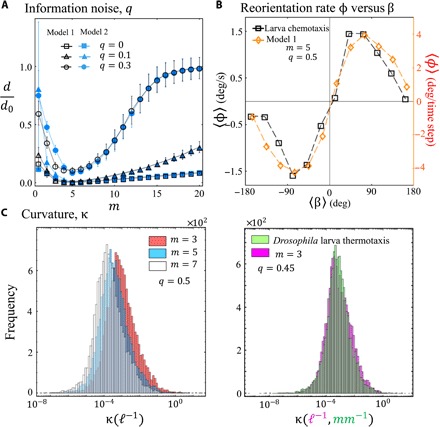
Effects of noise on performance and quantitative comparison with experiments. (**A**) Effect of noise *q* on the efficiency of reaching the target as measured by *d*/*d*_0_, as a function of the agents’ individual capability *m*. *N* = 101 and *d*_0_ = 10^4^*ℓ*. (**B**) Dependence of reorientation rate φ ≡ *d*θ/*dt* on the bearing angle β (Fig. 2A) for experimental larva chemotaxis trajectories (*25*) and our model with *m* = 5 and *q* = 0.5. Simulation averages are over 10^3^ trajectories with random initial conditions starting near the target for *n* = 10^2^ time steps. (**C**) Distribution of curvature values form trajectories of our model 1 (left panel) for the parameters shown and illustrative comparison with empirical larva thermotaxis (right). *N* = 101, *s* = 2, and δ = π/2*N*.

To demonstrate the robustness of our main findings to changes in the model, Figs. 2 to 4 present results using two distinct implementations. Figure 2 defines the physical quantities (Fig. 2A) that determine the motion updates (Fig. 2B) and hence the distance *d* from the target after a given time (Fig. 2C). At time *t*, the position is (*x*_*t*_, *y*_*t*_) in the plane. The target is fixed at (*x*_*T*_, *y*_*T*_). The system moves with a velocity ${\vec{v}}_{t}$, which makes an angle θ_*t*_ with the horizontal. The vector ${\vec{T}}_{t}$, pointing from the system to the target, determines the ideal trajectory. Hence, the winning action is the one that better aligns the vectors $\vec{v}$ and $\vec{T}$. Our first implementation (version model 1) compares the alignment of these vectors before and after the rotation at the same position in space. Let Ω_*t* −1_ be the angle between ${\vec{v}}_{t-1}$ and ${\vec{T}}_{t-1}$ at time *t* − 1. Once each of the agents independently decides on an action, the aggregate of these actions rotates the system. Before it moves in the new direction, the new vector velocity now makes an angle Ω′ with the target vector. The difference between these two angles, ΔΩ = Ω′ − Ω_*t*−1_, is used to discern whether a specific action is a winning one. Both angles are always taken as positive so that the ideal action would make Ω′ smaller than Ω_*t*−1_ (i.e., better alignment after rotation). If ΔΩ > 0, then the action that made Ω′ larger is the losing action. If ΔΩ < 0, then the action that made Ω′ smaller is the winning action. If ΔΩ = 0 (i.e., direction unchanged) and Ω_*t*−1_ ≠ 0 (and thus Ω′ ≠ 0), then the action that made Ω′ smaller is the winning action. Last, if ΔΩ = 0 and Ω_*t*−1_ = Ω′ = 0 (i.e., already aligned), then both actions win. The second implementation (version model 2) looks at the instantaneous alignment of $\vec{v}$ and $\vec{T}$ at the current point in space and time. This is attained through the bearing angle β_*t*_, which opens from ${\vec{T}}_{t}$ to ${\vec{v}}_{t}$ (see Fig. 2A). |β| = Ω, since Ω is defined as positive. The winning action is decided by the sign of β_*t*_: If β_*t*_ > 0, then there is an excess of agents choosing action +1 and hence action −1 is the winning one. On the contrary, if β_*t*_ < 0, then there is an excess of agents choosing action −1 and thus action +1 is the winning action. When β_*t*_ = 0, the wining action is either. Table 1 schematically enumerates the steps our navigational algorithms execute to advance the entity’s way to the target.

**Table 1:** Steps executed by our navigational algorithms model 1 (left) and model 2 (right).

**Model 1**	**Model 2**
While 1 ≤ *t* ≤ *n*	While 1 ≤ *t* ≤ *n*
1. Entity at (*x*_*t*−1_, *y*_*t*−1_) and Ω_*t*−1_	1. Entity at (*x*_*t*_, *y*_*t*_) and β_*t*_
2. Agents choose action ±1	2. Winning action decided (see Fig. 2B)
3. Entity rotates → new orientation is Ω′	3. Agents choose action ±1
4. Winning action decided (see Fig. 2B)	4. Entity rotates
5. Entity translates	5. Entity translates
6. *t* ← *t* − 1	6. *t* + 1 ← *t*
7. Go to 1	7. Go to 1

## RESULTS

### Efficiency

Figure 2C shows that the entity is remarkably efficient in reaching its target when its individual components are neither too capable nor too incapable, i.e., *m* has an intermediate value. It shows the ratio of the final system-target separation *d* to the initial separation *d*_0_ (see inset), as a function of each agent’s capability *m* for version model 1 (black symbols) and model 2 (blue symbols). We set the total simulation time equal to *n* time steps with *n* = *d*_0_/*ℓ*, so the system would reach the target at *t* = *n* (i.e., *d*/*d*_0_ = 0) if the alignment along the path was always perfect. For both model variants, there is a maximum in efficiency (i.e., minimum in *d*/*d*_0_), which is highly robust to changes in the distance of the target, damage of the *N* components, and substitution or loss. This finding of a switch between increasing and decreasing efficiency with increasing *m* offers an answer to the question of when and why evolution in natural systems switched between decentralized (e.g., larvae) and centralized (e.g., higher species) designs. By contrast, if the entity was executing a purely random walk, then the value of *d*/*d*_0_ would be of order 1 for all *m* and hence off the vertical scale.

The counterintuitive conclusion from Fig. 2 that increasing the capability *m* of the individual components (agents) eventually decreases the overall efficiency can be explained and described mathematically by considering the correlations in the strategy pool via an extension of the crowd-anticrowd theory (*33*–*36*). If *m* is large, then the number of possible strategies ($2^{2^{m}}$) is large, and hence the probability that any two agents hold and use the same strategy at any given time is small. Hence, the agents will tend to act independently. This means that, as *m* increases, the trajectory of the entity becomes increasingly like a random walk, and hence the ability to reach a given target decreases, i.e., the deviation *d* after a given number of time steps *n* will increase as *m* → ∞. By contrast, when *m* is small, the number of possible strategies is small, and hence the probability that any two agents hold and use the same strategy at any given time is large. The agents are now strongly correlated and so effectively act as a crowd. Just as in a canoe, if all the occupants suddenly paddle on the same side, then the entity will turn through large angles at each time step as well. These large fluctuations in angle (Fig. 3) make the entity waste time steps by successively overcorrecting previous overcorrections (*33*–*36*). Hence, as *m* decreases, the entity’s ability to reach a given target decreases, i.e., the deviation *d* after a given number of time steps *n* will increase as *m* → 0. Figure 3 demonstrates the accuracy of our analytic crowd-anticrowd theory in quantifying the fluctuations in the orientation angle at each time step as a function of each agent’s capability *m*.

### Theoretical approach

We have derived an accurate mathematical formula for *d*/*d*_0_ (pink line in Fig. 2C) by generalizing the crowd-anticrowd theory (*35*, *36*) up to second order in the angle covariance and fourth order in angle fluctuations

$$\begin{array}{c} \frac{d}{d_{0}}\approx [\frac{\sigma_{\theta }^{2}}{n}+\sigma_{\theta }^{4}(\frac{1}{12}-\frac{1}{6n})+\frac{2}{n^{2}}(1-\frac{\sigma_{\theta }^{2}}{3}+\frac{\sigma_{\theta }^{4}}{30})C+\\ {\frac{2}{n^{2}}(\frac{1}{6}-\frac{\sigma_{\theta }^{2}}{30}+\frac{\sigma_{\theta }^{4}}{420})C^{2}]}^{1/2} \end{array}$$ (1)

where $C=\sum_{j=1}^{n-1}\text{cov}[\{\theta_{i}\},\{\theta_{i+j}\}]$, which can be calculated by simulation data but is well approximated by the algebraic form proportional to *m*^α^ (see the Supplementary Materials). For the limiting case of *n* → ∞ (i.e., the target is far away), the accuracy ratio is reduced to $d/d_{0}\approx \sigma_{\theta }^{2}/\sqrt{12}$ and shown in the green curve in Fig. 2C. The angular fluctuations over time σ_θ_ are given by (see the Supplementary Materials)

$$\sigma_{\theta }={\left(\frac{1}{2}\sigma_{CA}^{2}+\text{cov}[\{\theta_{i}\},\{\theta_{i+1}\}]\right)}^{1/2}$$ (2)

where the formula for the fluctuations at each time step $\sigma_{CA}^{2}$ is derived in the Supplementary Materials and its accuracy is shown explicitly in Fig. 3. For large *m*, the full form of σ_θ_ (Fig. 3 orange curve) in Eq. 2 shows good agreement. For small to intermediate *m*, the analytic formula agrees well with the simulation data and captures the optimal point at *m* = 5. This allows us to provide an approximate formula for the optimum capability *m*_*o*_ of a system’s components or members such that the entity will come as close as possible to its desired target

$$\frac{\sqrt{N}}{2^{(m_{o}+1)/2}}{\left(1+\frac{1}{3}(1-2^{-2(m_{o}+1)})\right)}^{\frac{1}{2}}=1$$ (3)

where the desired *m*_*o*_ is the solution to this equation. This estimate is general for binary agent resource models and can be used as a first approximation of the optimum capability for specific implementations. While this uses the lower-bound analytic expression in Fig. 3, the corresponding result for the upper-bound differs trivially by an extra factor $\sqrt{2}$ (see the Supplementary Materials).

### Noise effects

Figure 4A shows that our main conclusions—and in particular, the appearance of an optimal agent capability *m*_*o*_—are remarkably robust to noise. This noise is introduced by a probability *q* that the actual outcome 0 or 1 gets corrupted before it is fed back to the agents. The depth of the *d*/*d*_*o*_ minimum, and hence the strength of the efficiency maximum, increases as the noise level *q* increases, while the value of the optimal capability *m*_*o*_ is insensitive to *q*, suggesting that Eq. 3 is universal. This effect highlights the greater capacity for error correction that a system which effectively balances crowd and anticrowd behavior (i.e., *m* = *m*_*o*_) will have. For smaller agent capability (*m* < *m*_*o*_), the overcorrecting behavior that produces a sharp zigzag-like trajectory, where the crowd outnumbers the anticrowd, becomes curvier and wider by the random information. This makes the system waste many more time steps to find a favorable alignment with the target (see the Supplementary Materials). Similarly, as *m* grows greater than *m*_*o*_, the randomness tends to destroy the small crowds formed due to the large strategy space, and the decision-making operation approaches more quickly that of a random process. Having agents receive the information of the winning group incorrectly with probability *q* at any given time step mimics disturbances in the signal reception or processing or a defective sensor whether biological or synthetic.

### Comparison with larval klinotaxis

While Fig. 1B shows visual commonality between *Drosophila* larvae trajectories and those of our model, we now make this comparison more quantitative. First, we consider the dependence of the turning rate ϕ (i.e., ϕ ≡ *d*θ/*dt*) on the bearing angle β (Fig. 2A). Empirically observed ϕ versus β measurements for chemotaxis dynamics (e.g., odor-driven) in *Drosophila* larvae and nematode *Caenorhabditis* *elegans* display a sinusoidal dependence (*18*, *25*). This finding supports the hypothesis that a proportional navigation method (*30*), which tends to preserve the line of sight angle fixed along the path, is likely used by these simple organisms when performing exploratory routines (*19*). As shown in Fig. 4B, our model version 1 captures the empirical sinusoidal dependence and magnitude well (*25*). Although we use *m* = 5 and *q* = 0.5, a sinusoidal pattern also arises for many other parameter choices [see the Supplementary Materials and (*37*)]. The noise level *q* tends to control the amplitude of the pattern, with smaller amplitudes associated with higher levels of randomness. This effect is consistent with the observations in larvae organisms, which show that they make turning decisions on a stimulus gradient against the optimal conditions about 40 to 45% of the time (*16*, *26*). The organisms do not immediately turn around when heading toward bad conditions; they eventually turn away from improving conditions. This is actually good in a realistic ecological context, because having randomness on top of purposeful motion is important for avoiding “traps” and/or finding improved conditions that could be on the other side of bad conditions (*16*, *26*). This empirically testable prediction of our model may help address open questions regarding how animals adapt to or exploit randomness.

Second, we look at the curvature κ at equally spaced time steps for 200 individual trajectories of duration 10^4^ steps, for different model parameters, and for the *Drosophila* larvae organisms undergoing positive thermotaxis. Figure 4C shows the resulting distributions where the points in the experimental path are taken to be 5 s apart, while for the model, they are at 10–time step intervals. Calculations for single-step spacing yield similar conclusions (see the Supplementary Materials). As shown, the peak of the distribution tends to shift to smaller values when the memory grows (Fig. 4C, left panel), since the trajectories tend to present more turns, as shown in Fig. 1B and fig. S1. We found a favorable comparison between the distributions of curvature values of the organism and our model for some specific parameters. The right panel of Fig. 4C shows an example for *m* = 3 and randomness of *q* = 0.45 that is in agreement with the observations for the organism’s behavior. To further analyze the relationship between the distributions, we carry out a Kolmogorov-Smirnov (KS) goodness-of-fit test. The comparison shown in Fig. 4C yields an average *P* value of 0.3621. Overall, we found that a combination of noise values between 0.45 and 0.5 and agent capabilities of 3 and 4 provide consistently high *P* values (see the Supplementary Materials). This is surprising given the high degree of randomness in both the model and the organism. By contrast, in a comparison with a null model where agents flip a coin to decide between actions ±1, the test gives a *P* value of 10^−13^. Additional tests results are presented in the Supplementary Materials.

## DISCUSSION

We acknowledge the limitations of our quantitative analysis. Our goal is not to do a rigorous comparison since we are not claiming to propose a detailed model for the larva organism. In contrast, our model is minimal and should be seen very much as a favorable prototype. Its value comes precisely from the fact that, despite its simplicity, it is able to produce nontrivial trajectories that are consistent with those performed by the organism. Individual trajectories, in both the model and the organism, are built from a large amount of randomness, and hence, we consider that an aggregated statistic is appropriate to establish a comparison between them. Our assumptions regarding the time and distance units (i.e., assuming that *ℓ* is comparable to millimeters and that time steps are comparable to seconds) are representative and used only as an illustrative tool. The main conclusions of this paper do not directly depend on these choices.

Last, we note that, because the trajectories of the decentralized entity are ultimately determined by the heterogeneity of the agents in our model, it is conceivable that our findings may shed some light on managing movement in individuals with significant limitations in coordination due to central nervous system injury or disease (*38*–*41*). Although entirely speculative at this stage, it is possible that, instead of trying to re-establish central control, one might instead be able to use the understanding of how the ecology of strategies held by the agents in Fig. 1 affects the range of movements to manage the ecology of disparate nerve and muscle elements—in such a way that uncontrolled movements (i.e., *d*/*d*_0_) are reduced, with the potential advantage that lower capability nerve and muscle elements (i.e., intermediate *m*) might work better (*38*–*41*). Similarly, our results can guide the development of new-generation technology of ingestible sensors to monitor and diagnose gastrointestinal health (*42*). Likewise, our results may help spark new ideas about the performance of autonomous vehicles having simpler components in a decentralized design, as in our larva model. This follows earlier suggestions that future system designs might usefully learn from nature’s own evolutionary solutions [see, for example, (*43*) and (*3*)]. Organisms such as *Drosophila* larvae harmonize the tasks of movement and turning through the collective output of individual segments of the body acting as a sensor and actuator, as mimicked by our model. Similarly, there is conceivably a real-world need for vehicles that can arrive “close enough” to some target, as in this paper, without needing very precise actions at each instance in time—and hence the vehicle could, following the results in this paper, be designed using simpler components than might otherwise be imagined. Although the lack of accuracy would not be appropriate for regular vehicle traffic, it might be acceptable for remote vehicle terrain exploration in hostile environments, where the overriding feature is to avoid centralized control, e.g., on the bottom of a deep ocean where central control may not be possible or in a conflict setting where a central control might be hacked or destroyed (*44*).

## MATERIALS AND METHODS

### Experimental design

Trajectories of the *Drosophila* larva organism were captured with high–pixel density video cameras with a resolution of 15 frames per second. The organisms were placed atop a 22 cm by 22 cm agar substrate while experiencing a one-dimensional thermal gradient ranging from 17.5° up to 22°C.

### Statistical analysis

The KS goodness-of-fit analysis that we used to obtain our results for the *P* values between the curvature distribution of the experiments and our model was carried out through the standard statistical package available in Wolfram Mathematica version 11.2. To compare the curvature distributions, we took 1000 random samples of sizes 500 and 1000 from each distribution and calculated the KS *P* value for each of them. The reported average *P* values are the average over all the tests of the individual random samples.

## Supplementary Material

http://advances.sciencemag.org/cgi/content/full/5/2/eaau5902/DC1

## SUPPLEMENTARY MATERIALS

Supplementary material for this article is available at http://advances.sciencemag.org/cgi/content/full/5/2/eaau5902/DC1 Section S1. Trajectory model Section S2. Trajectory model—External field Section S3. Turning rate, noise, and curvature Section S4. Theoretical approach Section S5. Crowd-anticrowd theory Fig. S1. Portion of trajectories in runs for different values of *m*, *q*, and δ for a target at (0, 10^4^*ℓ*), an initial location at (0, 0), and an initial direction θ = π/2. Fig. S2. Complete trajectories for a close reaching target. Fig. S3. Trajectories of runs of *d*_0_ time steps for different values of *m* (*N* = 101 and *s* = 2) when there is a drift of velocity of (0, −0.1), (0, 0.1), (0.1, 0), and (−0.1, 0). Fig. S4. Turning rates and noise effects for a collection of model trajectories with different parameters. Fig. S5. Curvature distribution calculations for our navigational model 1 and the larva organism. Fig. S6. Sum of covariance (C and $C^{2}$) calculated from simulation data as a function of memory *m*. Fig. S7. Schematics representation of the matrix Ψ for *m* = 2 and *s* = 2 in the Reduced Strategy Space (RSS). Fig. S8. Crowd-anticrowd theory against numerical simulations for the model organism as a function of the agent capability *m*, for *N* = 101 agents, *s* = 2, and δ = π/2*N*. Table S1. Statistical similarity between the curvature distribution of the larva organism and our model 1 using the KS test.

## References

[1] Robotic Rules of the Road (The Economist, print edition, 2018), p. 67.

[2] Konstantakopoulos I. C., Ratliff L. J., Jin M., Shankar Sastry S., Spanos C. J., A robust utility learning framework via inverse optimization. IEEE Trans. Control Syst. Technol. 26, 954–970 (2018).

[3] C. Lo, K. Bhardwaj, R. Marculescu, An autonomous and adaptive bacteria-based drug delivery system, in *Proceedings of the Third ACM International Conference on Nanoscale Computing and Communication*, New York, NY, USA, 28 to 30 September 2016.

[4] Zhao L., Yang G., Wang W., Chen Y., Huang J. P., Ohashi H., Eugene Stanley H., Herd behavior in a complex adaptive system. Proc. Natl. Acad. Sci. U.S.A. 108, 15058–15063 (2011). 21876133 10.1073/pnas.1105239108 PMC3174612

[5] Manrique P. D., Zheng M., Cao Z., Johnson D., Hui P. M., Johnson N. F., Subsecond tsunamis and delays and delays in decentralized electronic systems. Electronics 6, 80 (2017).

[6] Manrique P. D., Johnson D. D., Johnson N. F., Using competition to control congestion in autonomous drone systems. Electronics 6, 31 (2017).

[7] J. Cartlidge, C. Szostek, M. D. Luca, D. Cliff, Too fast too furious: Faster financial-market trading agents can give less efficient markets, in *4th International Conference on Agents and Artificial Intelligent, Agents (ICAART-2012)*, J. Filipe, A. Fred, Eds. (SciTePress, 2012), vol. 2, pp. 126–135.

[8] Johnson N. F., Zhao G., Hunsader E., Qi H., Johnson N., Meng J., Tivnan B., Abrupt rise of new machine ecology beyond human response time. Sci. Rep. 3, 2627 (2013). 24022120 10.1038/srep02627 PMC3769652

[9] Johnson N. F., To slow or not? Challenges in subsecond networks. Science 355, 801–802 (2017). 28232541 10.1126/science.aai8618

[10] Floreano D., Wood R. J., Science, technology and the future of small autonomous drones. Nature 521, 460–466 (2015). 26017445 10.1038/nature14542

[11] Rubenstein M., Cornejo A., Nagpal R., Programmable self-assemblt in thousand-robot swarm. Science 345, 795–799 (2014). 25124435 10.1126/science.1254295

[12] Zhang W. H., Chen A., Rasch M. J., Wu S., Decentralized multisensory information integration in neural systems. J. Neurosci. 36, 532–547 (2016). 26758843 10.1523/JNEUROSCI.0578-15.2016 PMC4710773

[13] Xiang Y., Yuan Q., Vogt N., Looger L. L., Jan L. Y., Jan Y. N., Light-avoidance-mediating photoreceptors tile the *Drosophila* larval body wall. Nature 468, 921–926 (2010). 21068723 10.1038/nature09576 PMC3026603

[14] Liu L., Yermolaieva O., Johnson W. A., Abboud F. M., Welsh M. J., Identification and function of thermosensory neurons in *Drosophila* larvae. Nat. Neurosci. 6, 267–273 (2003). 12563263 10.1038/nn1009

[15] Kwon Y., Shim H. S., Wang X., Montell C., Control of thermotactic behavior via coupling of a TRP channel to a phospholipase C signaling cascade. Nat. Neurosci. 11, 871–873 (2008). 18660806 10.1038/nn.2170

[16] Luo L., Gershow M., Rosenzweig M., Kang K., Fang-Yen C., Garrity P. A., Samuel A. D. T., Navigational decision-making in *Drosophila* thermotaxis. J. Neurosci. 30, 4261–4272 (2010). 20335462 10.1523/JNEUROSCI.4090-09.2010 PMC2871401

[17] Klein M., Krivov S. V., Ferrer A. J., Luo L., Samuel A. D. T., Karplus M., Exploratory search during directed navigation in *C. elegans* and *Drosophila* larva. eLife 6, e30503 (2017). 29083306 10.7554/eLife.30503 PMC5662291

[18] Iino Y., Yoshida K., Parallel use of two behavioral mechanisms for chemotaxis in *Caenorhabditis elegans*. J. Neurosci. 29, 5370–5380 (2009). 19403805 10.1523/JNEUROSCI.3633-08.2009 PMC6665864

[19] Martinez D., Klinotaxis as a basic form of navigation. Front. Behav. Neurosci. 8, 275 (2014). 25177280 10.3389/fnbeh.2014.00275 PMC4132367

[20] Berni J., Pulver S. R., Griffith L. C., Bate M., Autonomous circuitry for substrate exploration in freely moving *Drosophila* larvae. Curr. Biol. 22, 1861–1870 (2012). 22940472 10.1016/j.cub.2012.07.048 PMC4082562

[21] Gomez-Marin A., Stephens G. J., Louis M., Active sampling and decision making in *Drosophila* chemotaxis. Nat. Commun. 2, 441 (2011). 21863008 10.1038/ncomms1455 PMC3265367

[22] Gomez-Marin A., Louis M., Active sensation during orientation behavior in the *Drosophila* larva: More sense than luck. Curr. Opin. Neurobiol. 22, 208–215 (2012). 22169055 10.1016/j.conb.2011.11.008

[23] Lahiri S., Shen K., Klein M., Tang A., Kane E., Gershow M., Garrity P., Samuel A. D. T., Two alternative motor programs drive navigation in *Drosophila* larva. PLOS ONE 6, e23180 (2011). 21858019 10.1371/journal.pone.0023180 PMC3156121

[24] Kane E. A., Gershow M., Afonso B., Larderet I., Klein M., Carter A. R., de Bivort B. L., Sprecher S. G., Samuel A. D. T., Sensorimotor structure of *Drosophila* larva phototaxis. Proc. Natl. Acad. Sci. U.S.A. 110, E3868–E3877 (2013). 24043822 10.1073/pnas.1215295110 PMC3791751

[25] Gomez-Marin A., Louis M., Multilevel control of run orientation in *Drosophila* larval chemotaxis. Front. Behav. Neurosci. 8, 38 (2014). 24592220 10.3389/fnbeh.2014.00038 PMC3923145

[26] Klein M., Afonso B., Vonner A. J., Hernandez-Nunez L., Berck M., Tabone C. J., Kane E. A., Pieribone V. A., Nitabach M. N., Cardona A., Zlatic M., Sprecher S. G., Gershow M., Garrity P. A., Samuel A. D. T., Sensory determinants of behavioral dynamics in *Drosophila* thermotaxis. Proc. Natl. Acad. Sci. U.S.A. 112, E220–E229 (2014). 25550513 10.1073/pnas.1416212112 PMC4299240

[27] W. Bialek, R. de Ruyter van Steveninck, F. Rieke, D. Warland, *Spikes: Exploring the Neural Code* (MIT Press, 1999).

[28] L. F. Abbott, P. Dayan, *Theoretical Neuroscience: Computational and Mathematical Modelling of Neural Systems* (MIT Press, 2001).

[29] Tang W. J., Wu Q. H., Biologically inspired optimization: A review. Trans. Meas. Control 31, 495–515 (2009).

[30] Murtauh S. A., Criel H. E., Fundamentals of proportional navigation. IEEE Spectr. 3, 75–85 (1966).

[31] Huang W. M., Ding Z., Wang C. C., Wei J., Zhao Y., Purnawali H., Shape memory materials. Mater. Today 13, 54–61 (2010).

[32] Pilate F., Toncheva A., Dubois P., Raquez J.-M., Shape-memory polymers for multiple applications in the materials world. Eur. Polym. J. 80, 268–294 (2016).

[33] Challet D., Zhang Y.-C., On the minority game: Analytical and numerical studies. Physica A 256, 514–532 (1998).

[34] Savit R., Manuca R., Riolo R., Adaptive competition, market efficiency and phase transitions. Phys. Rev. Lett. 82, 2203–2206 (1999).

[35] Johnson N. F., Hui P. M., Crowd-Anticrowd theory of dynamical behavior in competitive, multi-agent autonomous systems and networks. J. Comput. Intell. Electron. Syst. 3, 256–277 (2014).

[36] Johnson N. F., Smith D. M. D., Hui P. M., Multi-agent complex systems and many-body physics. Europhys. Lett. 74, 923–929 (2006).

[37] Manrique P., Klein M., Li Y. S., Xu C., Hui P. M., Johnson N., Decentralized competition produces nonlinear dynamics akin to klinotaxis. Complexity 2018, 9803239 (2018).

[38] Witten T. M., Introduction to the theory of aging networks. Interdiscip. Top. Gerontol. 40, 1–17 (2015). 25341509 10.1159/000364922

[39] Wimble C., Witten T. M., Applications to aging networks, in aging and health: A systems biology perspective. Interdiscip. Top. Gerontol. 40, 18–34 (2015). 25341510 10.1159/000364925

[40] O. Sporns, *Networks of the Brain* (MIT Press, 2010).

[41] Bullmore E., Sporns O., The economy of brain network organization. Nat. Rev. Neurosci. 13, 336–349 (2012). 22498897 10.1038/nrn3214

[42] Mimee M., Nadeau P., Hayward A., Carim S., Flanagan S., Jerger L., Collins J., McDonnell S., Swartwout R., Citorik R. J., Bulović V., Langer R., Traverso G., Chandrakasan A. P., Lu T. K., An ingestible bacterial-electronic system to monitor gastrointestinal health. Science 360, 915–918 (2018). 29798884 10.1126/science.aas9315 PMC6430580

[43] Wehner M., Truby R. L., Fitzgerald D. J., Mosadegh B., Whitesides G. M., Lewis J. A., Wood R. J., An integrated design and fabrication strategy for entirely soft, autonomous robots. Nature 536, 451–455 (2016). 27558065 10.1038/nature19100

[44] Liu Z., Zhang Y., Yu X., Yuan C., Unmanned surface vehicles: An overview of developments and challenges. Annu. Rev. Control 41, 71–93 (2016).

