# Supplementary material for: Getting closer to the goal by being less capable
Source: Sci Adv. 2019 Feb 6;5(2):eaau5902. doi: 10.1126/sciadv.aau5902 (PMC6365121; doi:10.1126/sciadv.aau5902)
Supplement: http://advances.sciencemag.org/cgi/content/full/5/2/eaau5902/DC1 [file supp_5_2_eaau5902__index.html]

Science Advances | Science Advances

## Supplementary Materials

**This PDF file includes:**

- Section S1. Trajectory model
- Section S2. Trajectory model—External field
- Section S3. Turning rate, noise, and curvature
- Section S4. Theoretical approach
- Section S5. Crowd-anticrowd theory
- Fig. S1. Portion of trajectories in runs for different values of *m*, *q*, and δ for a target at (0, 104*ℓ*), an initial location at (0, 0), and an initial direction θ = π/2.
- Fig. S2. Complete trajectories for a close reaching target.
- Fig. S3. Trajectories of runs of *d*0 time steps for different values of *m* (*N* = 101 and *s* = 2) when there is a drift of velocity of (0, −0.1), (0, 0.1), (0.1, 0), and (−0.1, 0).
- Fig. S4. Turning rates and noise effects for a collection of model trajectories with different parameters.
- Fig. S5. Curvature distribution calculations for our navigational model 1 and the larva organism.
- Fig. S6. Sum of covariance ( C and C2) calculated from simulation data as a function of memory *m*.
- Fig. S7. Schematics representation of the matrix Ψ for *m* = 2 and *s* = 2 in the Reduced Strategy Space (RSS).
- Fig. S8. Crowd-anticrowd theory against numerical simulations for the model organism as a function of the agent capability *m*, for *N* = 101 agents, *s* = 2, and δ = π/2*N*.
- Table S1. Statistical similarity between the curvature distribution of the larva organism and our model 1 using the KS test.

Download PDF

**Files in this Data Supplement:**

- Adobe PDF - aau5902\_SM.pdf
