# Supplementary material for: Getting closer to the goal by being less capable
Source: Sci Adv. 2019 Feb 6;5(2):eaau5902. doi: 10.1126/sciadv.aau5902 (PMC6365121; doi:10.1126/sciadv.aau5902)
Supplement: http://advances.sciencemag.org/cgi/content/full/5/2/eaau5902/DC1 [file aau5902_SM.pdf]

Science Advances
AAAS

advances.sciencemag.org/cgi/content/full/5/2/eaau5902/DC1

Supplementary Materials for

Getting closer to the goal by being less capable

Pedro D. Manrique*, Mason Klein, Yao Sheng Li, Chen Xu, Pak Ming Hui, Neil F. Johnson

*Corresponding author. Email: p.manriquecharry@umiami.edu

Published 6 February 2019, *Sci. Adv.* **5**, eaau5902 (2019)
DOI: 10.1126/sciadv.aau5902

This PDF file includes:

Section S1. Trajectory model
Section S2. Trajectory model—External field
Section S3. Turning rate, noise, and curvature
Section S4. Theoretical approach
Section S5. Crowd-anticrowd theory
Fig. S1. Portion of trajectories in runs for different values of m, q, and δ for a target at $(0, 10^4\ell)$, an initial location at (0, 0), and an initial direction $\theta = \pi/2$.
Fig. S2. Complete trajectories for a close reaching target.
Fig. S3. Trajectories of runs of d_0 time steps for different values of m ($N = 101$ and $s = 2$) when there is a drift of velocity of (0, –0.1), (0, 0.1), (0.1, 0), and (–0.1, 0).
Fig. S4. Turning rates and noise effects for a collection of model trajectories with different parameters.
Fig. S5. Curvature distribution calculations for our navigational model 1 and the larva organism.
Fig. S6. Sum of covariance (C and C^2) calculated from simulation data as a function of memory m.
Fig. S7. Schematics representation of the matrix Ψ for $m = 2$ and $s = 2$ in the Reduced Strategy Space (RSS).
Fig. S8. Crowd-anticrowd theory against numerical simulations for the model organism as a function of the agent capability m, for $N = 101$ agents, $s = 2$, and $\delta = \pi/2N$.
Table S1. Statistical similarity between the curvature distribution of the larva organism and our model 1 using the KS test.

Supplementary Materials

Section S1. Trajectory model

Figure S1 shows portions of model trajectories for the parameters $s = 2$, $N = 101$ and different values of the capability m, information noise q and rotation range δ. For small m, the strategy pool is small and all the strategies are in play. The crowd effect is big in the sense that in each round the majority out-numbers the minority by much. The majority becomes the wrong action and then soon the opposite action becomes the majority and it is also the wrong action. The outcomes move through the history space (the graph corresponding to the history space) almost regularly (periodically). The effect is that the boat will move in a rather regular zigzag motion (mean-reverting) in a short period (or short wavelength) like moving LLRRLLRR...and at a big inclined angle (crowd effect) to the vertical. The zigzag motion makes the end point farther away from the target. For some intermediate optimal m, the crowd is anti-balanced by the anticrowd. The model organism moves in a most direct way towards the target (since N is odd, it is also going left and right, but at an angle closer to the vertical and thus rather in a rather straight path). In this optimal situation, the end point is nearest to the target and thus d/d_0 is a minimum. For large m, the strategy pool is huge and only a small portion of the strategies are in play. Some best-performing strategies are not in play. It leads to a rather random motion of the steering direction and the wiggling is of longer wavelength. This also leads to the end point to be farther away from the target.

Additional trajectories from our model in the neighborhood the target are shown in Fig. S2 for different values of m, $s = 2$ and $N = 101$. Each panel of Fig. S2(a) shows three trajectories for a specific value of m when the system starts at a particular point in the x-axis and $y = 0$ while the target is located at the point $(0, 100)$ in length step units. For $m = 1$ (top panel), the trajectories are shown to follow a zig-zag type of pattern given the small number of strategies (4 strategies) compared to the number of agents. As a consequence, each strategy is selected a similar number of times during the dynamics creating crowds of agents that make the same action and an anticrowd that make the opposite. These actions tend to cancel each other out and the dynamics is dictated by the remaining agents that, given the short memory length, rapidly and quasi-periodically alternate the output making the system advance in zig-zag. Consequently, the end point is farther away from the target. Medium length memories (Fig. S2(a) middle panel) result in trajectories that tend to be straighter improving the efficiency of the movement. Since N is odd, there is also a left-right movement but at a smaller Ω straightening the path. As shown, the end point is nearest to the target. For large memories (Fig. S2(a) bottom panel) the strategy pool is huge and only small portions of the strategies are in play. Consequently, some best-performing strategies are not in play leading to a rather random direction making the system hard to be put on track. This also leads to the end point to be farther away from the target. Figure S2(b) compares trajectories for three memory lengths when the system

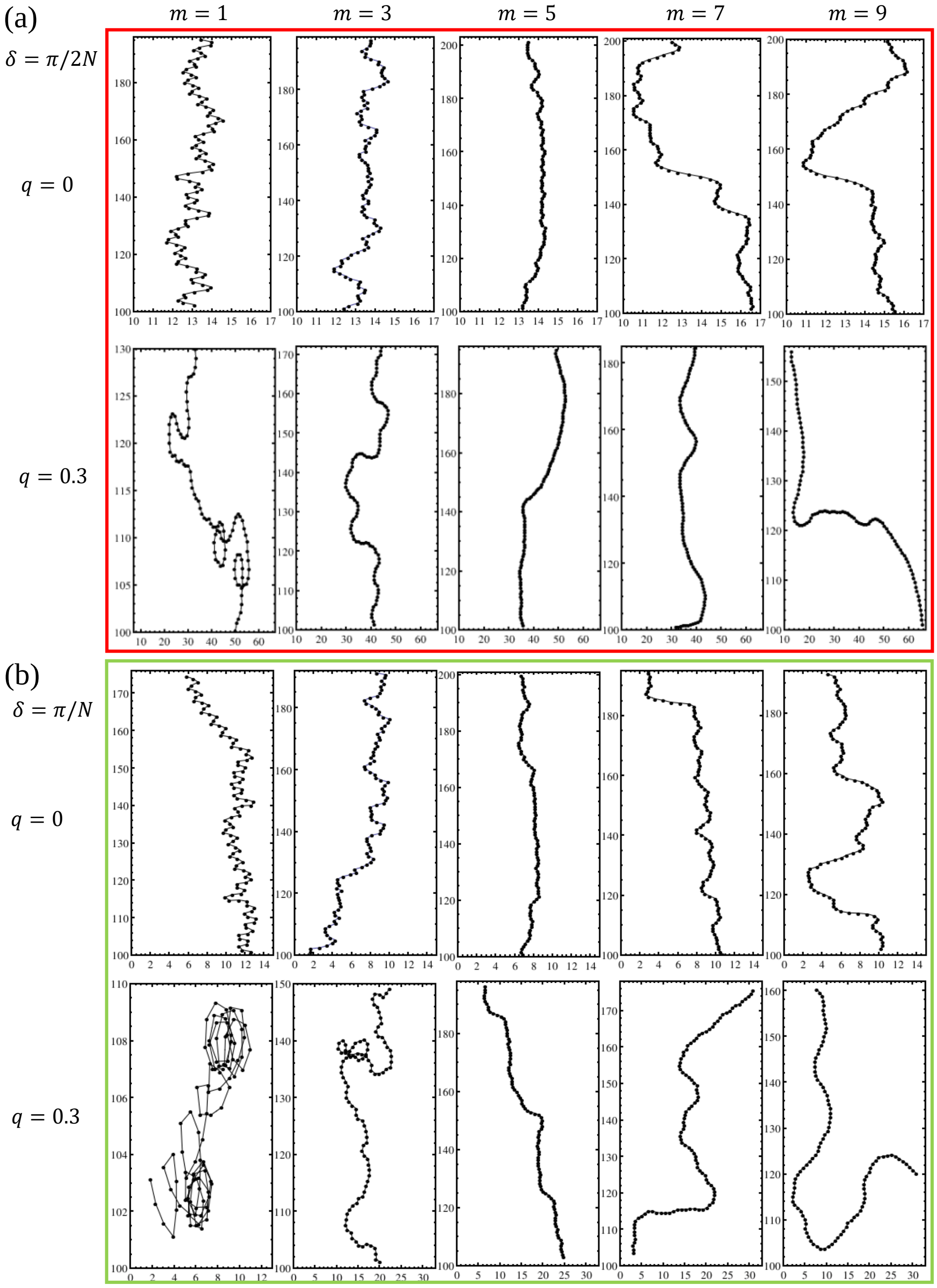

Fig. S1. Portion of trajectories in runs for different values of m, q and δ for a target at $(0, 10^4\ell)$, initial location at $(0, 0)$, and initial direction $\theta = \pi/2$. For small m and p, the zigzag motion is clearly observed. It is due to the crowd effect. For the optimal value of $m = 5$ where d/d_0 is smallest, the path is most directed towards the target. It is due to crowd-anticrowd effect. For large m, the path wanders around with turns at longer time intervals.

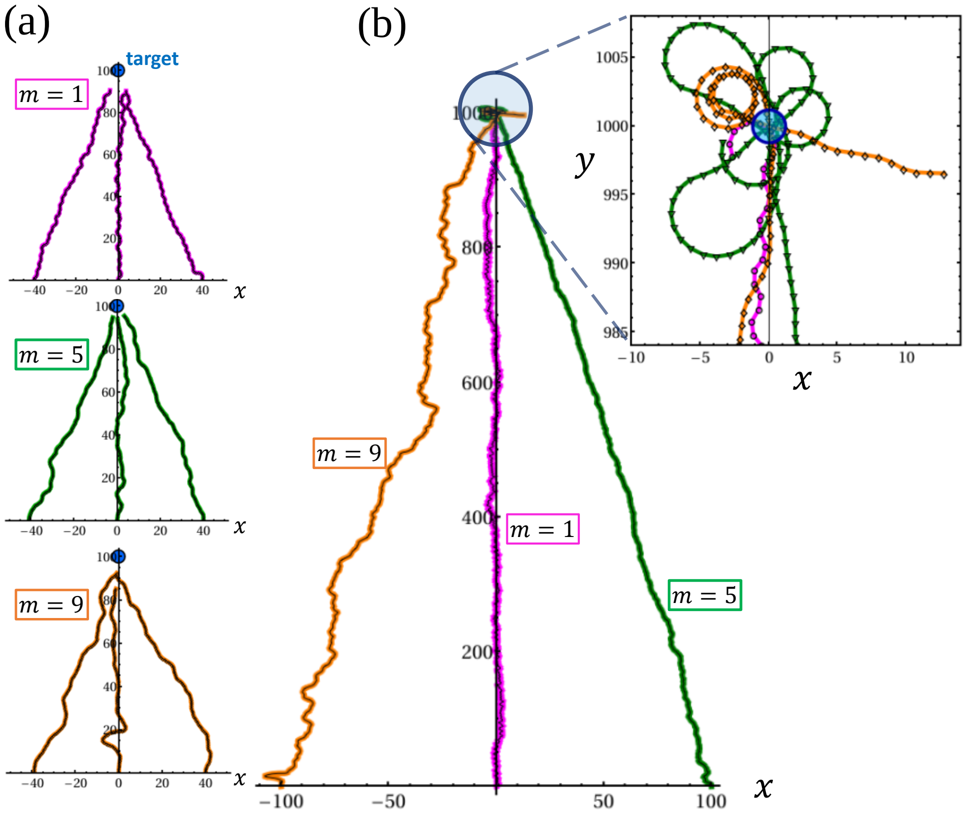

Fig. S2. Complete trajectories for a close reaching target. (a) Sample trajectories from our model moving from a specific point at the x axis and $y = 0$ toward a target located at the point $(0, 100)$ in distance units of one single step ℓ. Each panel shows three trajectories for a specific value of m, $m = 1$ (top), $m = 5$ (middle) and $m = 9$ (bottom). For each trajectory, the number of steps is such that the system would get to the target if traveling on a straight line. (b) Comparison of the trajectories for the three values of m of the A part, when the system runs for 100 extra steps. Inset shows a close-up of the target region. Other parameters are $s = 2$, $N = 101$ and $\delta = \pi/2N$.

make 100 extra steps. The inset shows the details in the vicinity of the target illustrating that medium memory (i.e. $m = 5$) is more efficient since it makes the larger number of loops around the target. This is followed by the large memory path (i.e. $m = 9$) with a few loops while the short memory case (i.e. $m = 1$) barely makes it to the target with no extra loops.

Section S2. Trajectory model - External field

Here we show that the agent capability parameter m that results in an optimal accuracy ($m = 4, 5$), is robust to the action of an external velocity field. For simplicity, we consider it to be constant in time for both magnitude and direction. We compare the model paths with the classical mechanics solution (labeled "swimming") where the system advances directly towards the target. Figure S3 shows our results when the velocity field acts in four contrasting directions at a magnitude of one tenth of the system's speed. The initial position is at $(-100, 0)$ and the target is at $(0, 1000)$. The step size for the system is 1. The run time is d_0, the steps that go from the initial position to the target directly in the absence of the velocity field. Note that in this case, the net movement of the system in a timestep is not 1. Without the field, the agents' collectively decide the angle and the system would move 1 unit in distance. But we need to add in the drift (which is a vector). The net movement is the sum of the two vectors, with a magnitude that is different from 1. The drift makes the moves slightly smaller or bigger than 1 unit. The line labelled 'swimming' is the solution to the classical mechanics problem of the same condition with the strategy of always swimming directly towards the target during the journey. The $m = 4$ case traces out the classical trajectory more closely, while other values of m give more wiggles. The bottom-right panel shows the statistics of d/d_0 as a function of m ($N = 101$ and $s = 2$) in the presence of a drift of $(0.1, 0)$ (squares) for runs of $d_0 = 10^4$ timesteps starting from $(0, 0)$ to a

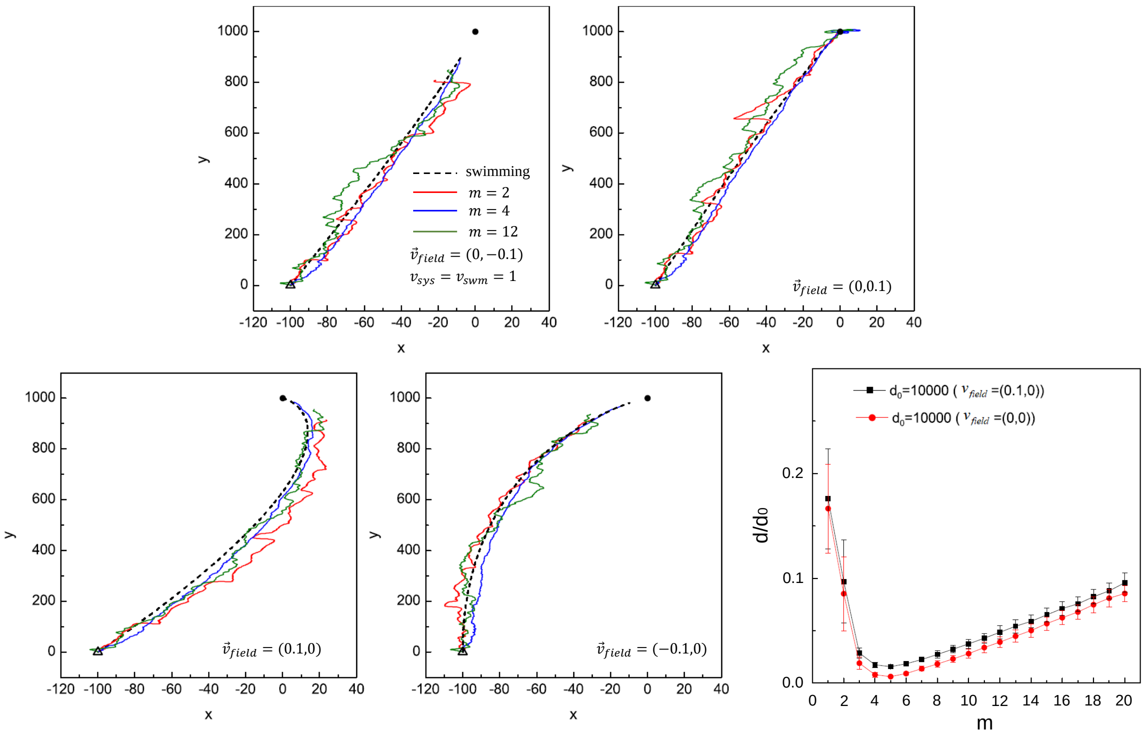

Fig. S3. Trajectories of runs of d_0 time steps for different values of m ($N = 101$ and $s = 2$) when there is a drift of velocity of $(0, -0.1)$, $(0, 0.1)$, $(0.1, 0)$, and $(-0.1, 0)$. The initial location is $(-100, 0)$ and the target is at $(0, 1000)$. Note that in this case, the actual movement of the boat in a time step is not exactly 1 unit. The bottom right panel shows d/d_0 as a function of m in the presence of a drift of $(0.1, 0)$ (squares) and no drift (circles) for runs of $d_0 = 10^4$ timesteps starting from (0, 0) to a target at (0, 10^4). Other parameters $N = 101$, $s = 2$, $\delta = \pi/2N$ and using winning rules given by model 1.

target at $(0, d_0)$ Note that in this case, the actual movement of the boat in a time step is not exactly 1 unit. The drift makes the moves slightly smaller or bigger than 1 unit. The results for the case with no drift (dots) are included for comparison. The optimal value of $m = 5$ is robust against the drift.

Section S3. Turning rate, noise and curvature

We look into the dependence of the turning rate ϕ, defined as the variation in the direction θ in a single timestep, with the bearing angle β, which is defined as the angle between the system's direction and the target vector $\vec{T}$ pointing always from the system to the target. Figure S4(a) schematically illustrates these defined quantities for a single step-trajectory. A navigational method known as proportional navigation, whose goal is to preserve the line of sight angle fixed while the system moves searching for the target, predicts a sinusoidal dependence between ϕ and β (see main paper). This pattern is also found in the crawling behavior of *Drosophila* larva and nematode *C. elegans* when performing chemotaxis (i.e. oddor-driven klinotaxis). Using a total of N individual trajectories, we test this dependence in our model organism following winning rules given by model 1 and model 2. The results, shown in Fig. S4(b), demonstrate that the sinusoidal dependence is followed by our model 1. We found a shift in the pattern when the system follows winning rules given by model 2 compared to model 1, indicating a larger tendency of the system rotate counter-clockwise. The effect of noise in the information, i.e. in a timestep, there is a small probability p that the winning action is announced incorrectly, is shown in Figure S4(c) for model 1. It is found that, as the randomness increases, the amplitude of the pattern decreases. Figure S4(d) shows the trajectory accuracy d/d_0 as a function of the probability of incorrect announcement p for different values of m. The target is at (0, d_0) and the initial location is at (0, 0). The run time is $d_0 = 10^4$ steps. The results show that $m = 5$ performs better for q up to about 0.45 where the noise washes out the effect of the memory m.

Figure S5 shows complementary results for the curvature distribution for our model 1. The top panel illustrates the effect of noise in the winner information for three values of agent capability m.

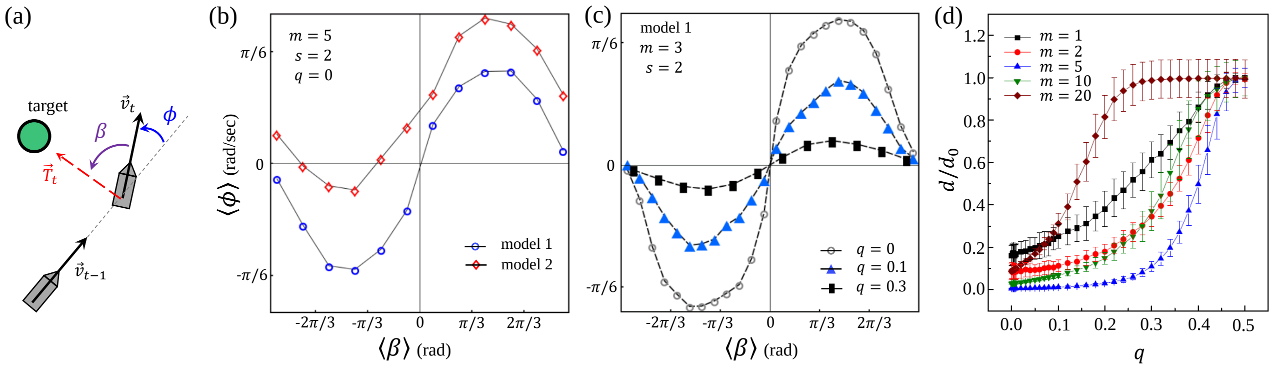

Fig. S4. Turning rates and noise effects for a collection of model trajectories with different parameters. (a) Schematics of strep-trajectory of our model organism in search for the target. The turning rate ϕ and the bearing angle β are defined. (b) Dependence of ϕ on β for the winning rules defined by model 1 (blue circles) and model 2 (red diamonds). (c) Dependence of ϕ on β for the winning rules defined by model 1 for different values of information noise q. Dependence of the average trajectory accuracy d/d_0 with the probability of incorrect announcement q for different values of m. The target is at $(0, d_0)$ and the initial location is at $(0, 0)$. The run time is $d_0 = 10^4$ steps for a population of $N = 101$ agents and $\delta = \pi/2N$.

For all values shown of m, mistaken information appear to affect the curvature distribution by shifting it to larger curvature values. This implies that the organism would be more likely to do small turns than remain at a straight path. As a consequence, entities experiencing large amounts of noise would lose efficiency in reaching the target, just as shown in Fig. S4(d). The bottom panel of Fig. S5 shows that entities with an optimal agent capability ($m_o = 5$) appears to be more resilient to these variations when compared to entities with other m values. This results from the robustness of the optimal agent capability to noise presented in Fig. 4(a) of the main paper. The right panel shows an illustrative comparison the curvature distribution for our model 1 ($m = 4$ and $q = 0.5$) and that for *Drosophila* larva thermotaxis exhibiting a reasonable agreement when we assume that the model displacement ℓ is comparable to 1 mm. For all cases, we have analyzed a total of 200 trajectories with a duration of 10^3 timesteps.

For this particular figure, the steps in the larva trajectories are taken to be spaced every 5 seconds while for the model we take them every 10 timesteps. We complement the illustrative comparison shown by doing a Kolmogorov-Smirnov test of the curvature distributions. Table S1 shows the average p-values from this particular test for different selection of parameters. We perform 1000 independent test using on each a random sample from the distribution of curvature values. The p-values in brackets are calculated using samples of 1000 entries while those without brackets are for sample sizes of 500 entries. The top section of table S1 shows the results when we analyze the organism trajectories every 5 seconds and the model trajectories every 10 timesteps. The bottom section shows the results when the organism trajectories are taken every half a second and the model trajectories every timestep. For both cases, we find consistently high values ($\bar{p} > 0.1$) for agent capabilities between 3 and 4, and erroneous information probability between 0.45 and 0.5.

Section S4. Theoretical approach

The observable d/d_0 can be calculated geometrically using the final position of the system (x_f, y_f), which in turn, is calculated by the history of directions $\{\theta_j\}$ with respect to the horizontal

$$d^2 = (d_0 - y_f)^2 + x_f^2, \tag{1}$$

$$x_f = \ell \sum_{j=1}^{n} \cos\theta_j, \quad y_f = \ell \sum_{j=1}^{n} \sin\theta_j \tag{2}$$

where d_0 is the initial system-target separation, ℓ is the length of each step and n is the number of timesteps. The square of the sums containing the sine and cosine functions can be rewritten in terms a double sum involving the cosine of the difference between the angles of two different timesteps

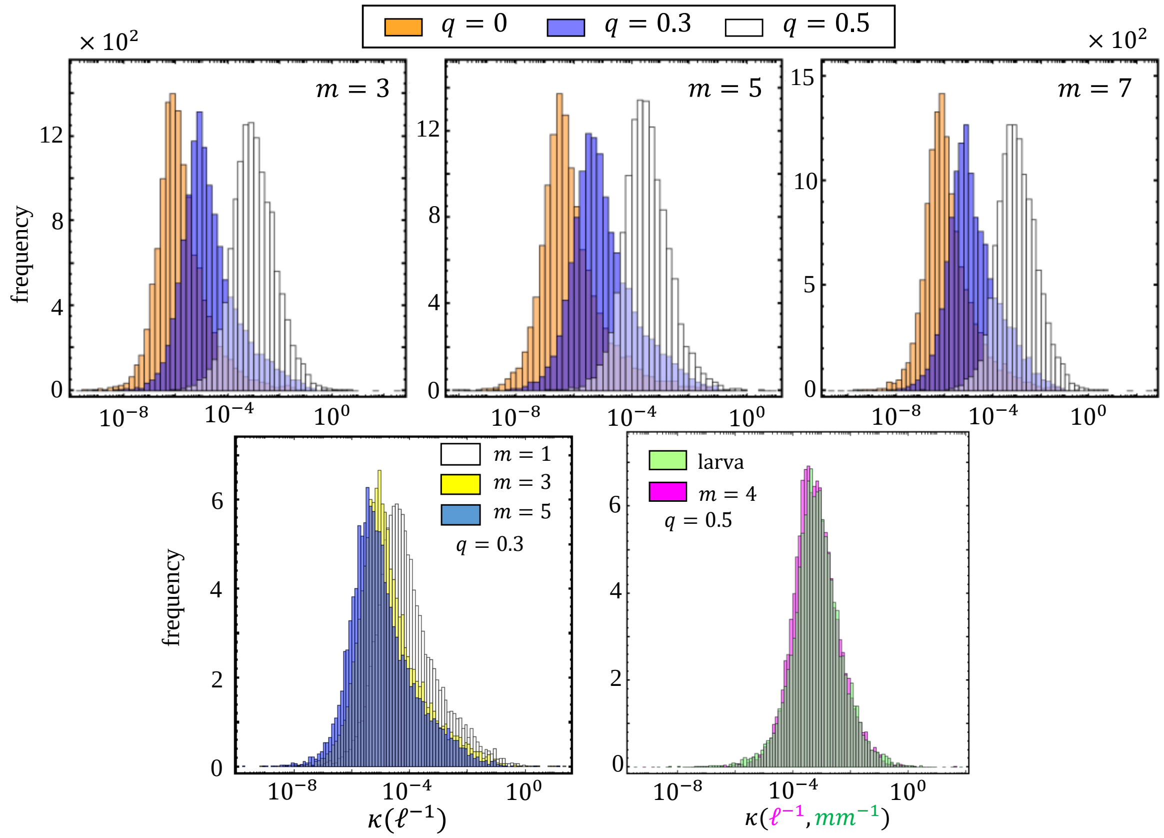

Fig. S5. Curvature distribution calculations for our navigational model 1 and the larva organism. Top: Each panel compares the model results for three values of probability of erroneous information q for $m = 3$ (left), $m = 5$ (center) and $m = 7$ (right). Bottom: The left panel compares for three different m values and for $q = 0.5$. The right panel compares the curvature from the experimental trajectories with the result of $m = 4$ and $q = 0.5$. All model results use the parameters $N = 101$, $s = 2$ and $\delta = \pi/2N$.

Table S1. Statistical similarity between the curvature distributions of the larva organism and our model 1 using the Kolmogorov-Smirnov test. Each entry is the average p-values for different combination of parameters. Values in brakets are calculated by testing random samples of 1000 curvature values, other are for a sample of 500. Values in bold are those that are greater than 0.1. Top section looks into larva trajectories whose steps are spaced 5 seconds and model steps 10 timesteps. Bottom section looks into trajectories spaced half a second and 1 timestep for the organism and model, respectively.

$q \mid m$	1	2	3	4	5
0.5	$10^{-5}(10^{-8})$	$10^{-3}(10^{-7})$	**0.3242(0.193)**	**0.3855(0.2878)**	0.0085(0.00028)
0.45	0.087(0.014)	**0.1577**(0.046)	**0.3621(0.245)**	0.046(0.006)	$10^{-5}(10^{-6})$
0.4	0.047(0.005)	$0.00562(10^{-6})$	$10^{-4}(10^{-8})$	$10^{-7}(10^{-13})$	$10^{-8}(10^{-15})$
0.5	$10^{-13}(0)$	$10^{-6}(10^{-10})$	**0.188**(0.091)	**0.405(0.31)**	0.01(0.00027)
0.45	$10^{-6}(10^{-10})$	0.01278(0.00054)	**0.332(0.212)**	0.063(0.0104)	$0.0007(10^{-6})$
0.4	0.043(0.0034)	0.0436(0.0038)	$0.0004(10^{-6})$	$10^{-7}(10^{-12})$	$10^{-8}(10^{-14})$

resulting

$$d^2 = d_0^2 - 2d_0\ell\sum_{j=1}^{n}\sin\theta_j + \frac{d_0^2}{n} + \frac{2d_0^2}{n^2}\sum_{i=1}^{n-1}\sum_{j=1}^{n-i}\cos(\theta_j - \theta_{j+i}) \tag{3}$$

After simplifying, the final exact form for d/d_0 is given by

$$\frac{d}{d_0} = \left(\frac{n+1}{n} - \frac{2}{n}\sum_{j=1}^{n}\sin\theta_j + \frac{2}{n^2}\sum_{i=1}^{n-1}\sum_{j=1}^{n-i}\cos(\theta_j - \theta_{j+i})\right)^{1/2} \tag{4}$$

where we have use $d_0 = n\ell$.

A mean field approach can be obtained by using the distribution of angles for each value of m. We change each step direction θ_j by its mean and fluctuations in the distribution, $\theta_j \to \bar{\theta} \pm \sigma_\theta$. We look at the case where the system lies initially at the origin $(0,0)$ and an initial direction of $\theta = \pi/2$, while the target is located at $(0, d_0)$. For this case the mean direction is $\pi/2$ for all the values of m. The last term of equation (4) can be expanded up to fourth order in σ_θ, and first order in covariance between $\{\theta_j\}$ and $\{\theta_{j+i}\}$, as follows

$$\begin{aligned}\sum_{i=1}^{n-1}\sum_{j=1}^{n-i}\cos(\theta_j - \theta_{j+i}) &\approx \frac{n^2-n}{2}\left(1 - \sigma_\theta^2 + \frac{\sigma_\theta^4}{6}\right) + \left(1 - \frac{\sigma_\theta^2}{3} + \frac{\sigma_\theta^4}{30}\right)\mathcal{C} \\ &+ \left(\frac{1}{6} - \frac{\sigma_\theta^2}{30} + \frac{\sigma_\theta^4}{420}\right)\mathcal{C}^2 \qquad (5) \\ \mathcal{C} &= \sum_{i=1}^{n-1}\mathrm{cov}[\{\theta_j\},\{\theta_{j+i}\}] \qquad (6)\end{aligned}$$

where $\mathrm{cov}[\{\theta_j\},\{\theta_{j+i}\}]$ is the covariance between angles $\{\theta_j\}$ and $\{\theta_{j+i}\}$. By adding the remaining terms and simplifying, the mean field expression becomes

$$\frac{d}{d_0} \approx \left[\frac{\sigma_\theta^2}{n} + \sigma_\theta^4\left(\frac{1}{12} - \frac{1}{6n}\right) + \frac{2}{n^2}\left(1 - \frac{\sigma_\theta^2}{3} + \frac{\sigma_\theta^4}{30}\right)\mathcal{C} + \frac{2}{n^2}\left(\frac{1}{6} - \frac{\sigma_\theta^2}{30} + \frac{\sigma_\theta^4}{420}\right)\mathcal{C}^2\right]^{1/2} \tag{7}$$

which is equation (1) in the main paper. The dependence of $\mathcal{C}^j$ on m is estimated by simulation data and found to depend algebraically on the system's capability as $\sim m^\alpha$, with $\alpha \approx 1.76$ for $j = 1$ and $\alpha \approx 3.5$ for $j = 2$, as presented in Figure S6. The equation (2) of the main paper is derived by looking at the change in the direction of motion generated by the collective action of the agents. The system's direction at time t, depends on the agent's collective actions together with the system's direction in the previous timestep

$$\theta_t = \theta_{t-1} + (n_{+1}[t] - n_{-1}[t])\delta \tag{8}$$

Therefore, the angular fluctuations σ_θ depend on the covariance between the directions one timestep apart and the fluctuations in the agent's collective action (see equation 2 in the main paper). The single-step covariance shows a dependence with the system capability of $\sim m^{3/2}$, while the latter can be estimated with the crowd-anticrowd theory for which we call it σ_{CA}.

Section S5. Crowd-Anticrowd theory

The implementation of the theory will broken down into two separate regimes: small m, corresponding to many more agents than available strategies and large m corresponding to the opposite case. Hence these two regimes are defined by the ratio of the number of strategies to agents being much less/greater than unity, and hence the strategy allocation matrix Ψ being densely/sparsely filled. The matrix Ψ is the symmetrized version of the allocation matrix Ω, ($\Psi = 1/2(\Omega + \Omega^T)$), which describes the distribution of strategies among the N individual agents. If this strategy allocation is fixed from the beginning of the game, then it acts as a quenched disorder in the system. The rank of Ω is given by

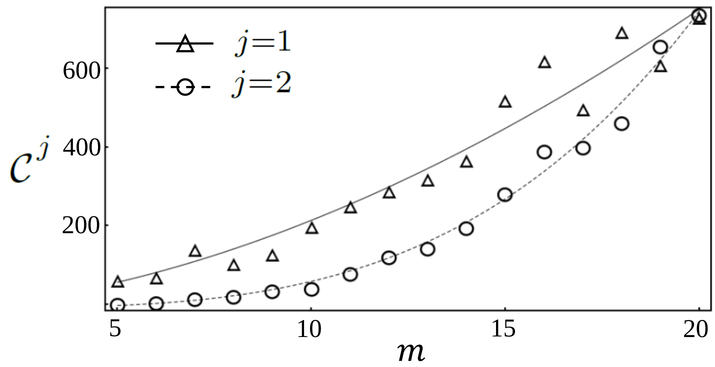

Fig. S6. Sum of covariance ($\mathcal{C}$ and $\mathcal{C}^2$) calculated from simulation data as a function of memory m. A total of 500 trajectories are used for each data point generated for different initial conditions of strategy distributions and winning histories. Curves are polynomial fits to the simulation data. Other parameters $s = 2$, $N = 101$ and $\delta = \pi/2N$.

the number of strategies s that each agent holds. For example, for $S = 3$ the element $\Omega_{i,j,k}$ gives the number of agents assigned strategy i, then strategy j, and then strategy k, in that order. Hence

$$\sum_{i,j,k,...}^{X} \Omega_{i,j,k,...} = N \tag{9}$$

where the value of X represents the number of distinct strategies that exist within the strategy space chosen: $X = 2^{2^m}$ in the Full Strategy Space (FSS), and $X = 2.2^m$ in the subset of strategies known as the Reduced Strategy Space (RSS), where any pair within this subset is either anticorrelated or uncorrelated. If we work in the RSS the directional change between two consecutive timesteps (step-rotation) can be written by summing over all the strategies as follows

$$n_{+1}[t] - n_{-1}[t] \equiv \sum_{R=1}^{2P} a_R^{\mu[t]} n_R^{\underline{S}[t]} \tag{10}$$

where $P = 2^m$. The quantity $a_R^{\mu[t]} = \pm 1$ is the response of strategy R to the history bit-string μ at time t. The quantity $n_R^{\underline{S}[t]}$ is the number of agents using strategy R at time t. The superscript $\underline{S}[t]$ is a reminder that this number of agents will depend on the strategy score at time t. Given that $a_R^{\mu[t]}$ and $n_R^{\underline{S}[t]}$ are uncorrelated, and that for competitive games there is no a priori best strategy, and assuming that the strategies are being distributed fairly, the average number of playing each strategy is approximately equal, $\left\langle n_R^{\underline{S}[t]} \right\rangle_t = \left\langle n_{\overline{R}}^{\underline{S}[t]} \right\rangle_t$, where the overline in the subindex $\overline{R}$ indicates the number of agents playing the anticorrelated strategy to R. With this in mind and using the exact result of $a_R^{\mu[t]} = -a_{\overline{R}}^{\mu[t]}$, the sum over the agents actions is zero as well as the average step rotation. Consequently, the variance of the step-rotation can be written as the square of the step-rotation as follows

$$\sigma_\Psi^2 = \sum_{R,R'=1}^{2P} \left\langle a_R^{\mu[t]} n_R^{\underline{S}[t]} a_{R'}^{\mu[t]} n_{R'}^{\underline{S}[t]} \right\rangle_t$$

In the case that the system visits all possible histories equally, the double sum can usefully be broken down into three parts, based on the correlations between the strategies: $\underline{a_R}.\underline{a_{R'}} = P$ (fully correlated), $\underline{a_R}.\underline{a_{R'}} = -P$ (fully anti-correlated), and $\underline{a_R}.\underline{a_{R'}} = 0$ (fully uncorrelated) where $\underline{a_R}$ is a vector of dimension P with R'th component $a_R^{\mu[t]}$. This decomposition is exact in the RSS in which we are working. Again we note that if all histories are not equally visited, yet some subset are, then this averaging can be carried out over the restricted subspace of histories. After simplifying, the equal-

histories case yields

$$\sigma_\Psi^2 \equiv \left\langle \sum_{R=1}^{P} \left(n_R^{S[t]} - n_{\overline{R}}^{S[t]} \right)^2 \right\rangle_t \tag{11}$$

The values of $n_R^{S[t]}$ and $n_{\overline{R}}^{S[t]}$ for each R will depend on the precise form of Ψ. We now proceed to consider the ensemble-average over all possible realizations of the strategy allocation matrix Ψ. The ensemble-average is denoted as $\langle ... \rangle_\Psi$, and for simplicity the notation $\langle \sigma_\Psi^2 \rangle_\Psi = \sigma_{CA}^2$ is defined. This ensemble-average is performed on either side of Equation (11)

$$\sigma_{CA}^2 = \left\langle \left\langle \sum_{R=1}^{P} \left(n_R^{S[t]} - n_{\overline{R}}^{S[t]} \right)^2 \right\rangle_t \right\rangle_\Psi \tag{12}$$

yielding the variance in the step-rotation. The next step into an analytic evaluation of equation 12 is to relabel the strategies form a decimal form R into a virtual-point ranking K. Thus, the specific identity of the 'K'th highest-scoring strategy', as well as $n_R^{S[t]}$, changes frequently in time. Therefore, it is convenient to shift the focus from the time-evolution of the virtual strategy scores $S_R[t]$, to the time-evolution of the virtual points of the K'th highest scoring strategy $S_K[t]$. Thus $K = 1$ denotes the highest scoring strategy position, $K = 2$ is the second highest and so on, assuming no strategy-ties. If all strategies start with score equal to zero, the anticorrelated strategies appear as the mirror-image, i.e., $S_K = -S_{\overline{K}}$. Hence, equation 12 can be rewritten as

$$\sigma_{CA}^2 = \left\langle \left\langle \sum_{K=1}^{P} \left(n_K^{S[t]} - n_{\overline{K}}^{S[t]} \right)^2 \right\rangle_t \right\rangle_\Psi \tag{13}$$

The advantage of this shift is that the quantities $n_K^{S[t]}$ and $n_{\overline{K}}^{S[t]}$ will fluctuate relatively little in time. The reason is that since agents play their highest score strategy, the relative ranking of these strategies and not the virtual score points becomes more relevant when calculating the number of agents playing a given strategy. This allows us to rewrite the number of agents playing the Kth ranked strategy at any timestep t, in terms of some constant value n_K plust a fluctuating term $\varepsilon_K[t]$. Hence, the variance becomes

$$\begin{aligned}
\sigma_{CA}^2 &= \left\langle \sum_{K=1}^{P} \left\langle \left[n_K + \varepsilon_K[t] - n_{\overline{K}} - \varepsilon_{\overline{K}}[t] \right]^2 \right\rangle_t \right\rangle_\Psi \qquad (14) \\
&= \left\langle \sum_{K=1}^{P} \left\langle \left[(n_K - n_{\overline{K}}) + (\varepsilon_K[t] - \varepsilon_{\overline{K}}[t]) \right]^2 \right\rangle_t \right\rangle_\Psi \\
&\approx \left\langle \sum_{K=1}^{P} \left\langle \left[n_K - n_{\overline{K}} \right]^2 \right\rangle_t \right\rangle_\Psi = \left\langle \sum_{K=1}^{P} \left[n_K - n_{\overline{K}} \right]^2 \right\rangle_\Psi
\end{aligned}$$

which involves no time dependence. The averaging over Ψ can be taken inside the sum and each of the terms can be rewritten using the joint probability distribution for n_K and $n_{\overline{K}}$, $P(n_K, n_{\overline{K}})$. Thus

$$\sigma_{CA}^2 = \sum_{K=1}^{P} \left\langle \left[n_K - n_{\overline{K}} \right]^2 \right\rangle_\Psi \tag{15}$$

$$= \sum_{K=1}^{P} \sum_{n_K=0}^{N} \sum_{n_{\overline{K}}=0}^{N} \left[n_K - n_{\overline{K}} \right]^2 P(n_K, n_{\overline{K}}) \tag{16}$$

The joint probability function $P(n_K, n_{\overline{K}})$ will depend on the ensemble of quenched $\{\Psi\}$ disorder which are being averaged over. In the 'crowded' limit of small m the matrices Ψ are nearly flat given that there are more agents than available strategies. Hence, the joint probability distribution will be

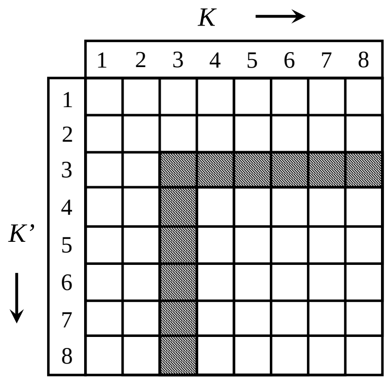

Fig. S7. Schematics representation of the matrix Ψ for $m = 2$ and $s = 2$ in the Reduced Strategy Space (RSS). For the crowded regime, the matrix is nearly flat and the number of agents playing the Kth ranked strategy is proportional to the number of shaded bins at that particular value of K.

sharply peaked around the n_K and $n_{\overline{K}}$ given by the mean for a flat quenched-disorder matrix Ψ, $\overline{n}_K$ and $\overline{n}_{\overline{K}}$. Therefore

$$\begin{aligned}\sigma^2_{CA} &= \sum_{K=1}^{P}\sum_{n_K=0}^{N}\sum_{n_{\overline{K}}=0}^{N}\left[n_K - n_{\overline{K}}\right]^2 \delta_{n_K,\overline{n_K}}\delta_{n_{\overline{K}},\overline{n_{\overline{K}}}} \\ &= \sum_{K=1}^{P}\left[\overline{n_K} - \overline{n_{\overline{K}}}\right]^2 \end{aligned} \quad (17)$$

I. Flat quenched disorder matrix Ψ, small m

Each element of Ψ has a mean of $N/(2P)^s$ agents per 'bin'. For the case of small m there is a densely-filled Ψ and the fluctuations in the number of agents per bin will be small compared to this mean value. For $s = 2$, the mean number of agents whose highest scoring strategy is the strategy occupying position K at timestep t, will therefore be given by summing the appropriate rows and columns of this quenched disorder matrix Ψ. Given that the matrix Ψ is flat, any re-ordering due to changes in the strategy ranking has no effect on the form of the matrix. Therefore the number of agents playing the K'th highest-scoring strategy, will always be proportional to the number of shaded bins at that K. (see Fig. S7 for $K = 3$ in the RSS). For example, the mean number of agents for the case of $K = 3$ becomes

$$\begin{aligned}\overline{n_{K=3}} &= N.\frac{1}{(2P)^2}\sum(\text{shaded bins}) \quad (18)\\ &= N.\frac{1}{64}.[(8-3)+(8-3)+1] = \frac{11}{64}N\end{aligned}$$

In the case of $s = 2$, $\overline{n_K}$ can be generalized to

$$\begin{aligned}\overline{n_K} &= N.\left(\left[1-\frac{(K-1)}{2P}\right]^2 - \left[1-\frac{K}{2P}\right]^2\right) \\ &= \frac{(2^{m+2}-2K+1)}{2^{2(m+1)}}N \end{aligned} \quad (19)$$

Likewise for $\overline{n_{\overline{K}}}$ the generalization is as follows

$$\overline{n_{\overline{K}}} = \frac{(2^{m+2}-2\overline{K}+1)}{2^{2(m+1)}}N = \frac{(2K-1)}{2^{2(m+1)}}N \quad (20)$$

where we have use the relation $\overline{K} = 2P - K + 1 \equiv 2^{m+1} - K + 1$. Using Equations 19 and 20 in

Equation 17 gives

$$\begin{aligned}\sigma_{CA}^2 &= \sum_{K=1}^{P}\left[\frac{(2^{m+2}-2K+1)}{2^{2(m+1)}}N - \frac{(2K-1)}{2^{2(m+1)}}N\right]^2 \\ &= \frac{N^2}{2^{2(2m+1)}}\sum_{K=1}^{P}[2^{m+1}-2K+1]^2 \\ &= \frac{N^2}{3\times 2^m}(1-2^{-2(m+1)})\end{aligned} \tag{21}$$

and hence

$$\sigma_{CA}^{\text{upperbound}} = \frac{N}{\sqrt{3}\times 2^{m/2}}(1-2^{-2(m+1)})^{\frac{1}{2}} \tag{22}$$

which is valid for small m. Here we have assumed that the game rules governing strategy ties do not upset the identical forms of the rankings in terms of highest virtual points and popularity. This tends to overestimate the size of the Crowds using high-ranking strategies, and underestimate the size of the Anticrowds using low-ranking strategies. Hence the Crowd-Anticrowd cancellation is underestimated, and consequently the result in equation 22 will overestimate the actual σ value meaning that it acts as an approximate upper-bound.

II. Non-flat quenched disorder matrix Ψ*, small* m

The appearance of a significant number of non-flat quenched disorder matrices Ψ in the ensemble, implies that the ranking in popularity of the strategies being used is not necessarily the same as the ranking in virtual points. In other words, we now have that $n_{K'} > n_{K''} > n_{K'''} > ...$, where the label K' need not equal 1, and K'' need not equal 2 etc.. Given this we introduce a new label $\{Q\}$ which will rank the strategies in terms of popularity, i.e.

$$n_{Q=1} > n_{Q=2} > n_{Q=3} > n_{Q=4} > ..., \tag{23}$$

where $Q = 1$ represents K', $Q = 2$ represents K'', etc.

With this in mind, we will return to the general form for the standard deviation of the step-rotation in Equation 15, but rewrite it slightly as follows:

$$\sigma_{CA}^2 = \frac{1}{2}\sum_{K=1}^{2P}\sum_{K'=1}^{2P}\left\{\sum_{n_K=0}^{N}\sum_{n_{K'}=0}^{N}[n_K - n_{K'}]^2 P(n_K, n_{K'})\right\} f_{K',\overline{K}}$$

where $f_{K',\overline{K}}$ is the probability that K' is the anticorrelated strategy to K (i.e. $\overline{K}$) and is hence given by $f_{K',\overline{K}} = \delta_{K',2P+1-K}$. A switch is now made to the popularity-labels $\{Q\}$. After relabelling, we obtain:

$$\sigma_{CA}^2 = \frac{1}{2}\sum_{Q=1}^{2P}\sum_{Q'=1}^{2P}\left\{\sum_{n_Q=0}^{N}\sum_{n_{Q'}=0}^{N}\left[n_Q - n_{Q'}\right]^2 P(n_Q, n_{Q'})\right\} \times f_{Q',\overline{Q}} \tag{24}$$

where $f_{Q',\overline{Q}}$ is the probability that the strategy with label Q' is anticorrelated to Q. It can be assumed that as a zeroth-order approximation the values of $n_{Q=1}$, $n_{Q=2}$, $n_{Q=3,...}$ etc. are still sharply peaked around their mean values obtained for the flat-matrix case. As before, we will label these values $\overline{n_Q}$ and $\overline{n_{Q'}}$ where the intrinsic dependence of Q on K has been dropped. Hence $P(n_Q, n_{Q'}) = \delta_{n_Q,\overline{n_Q}}\delta_{n_{Q'},\overline{n_{Q'}}}$ with $\overline{n_Q}$ and $\overline{n_{Q'}}$ given by the bin-counting method. Substituting in $P(n_Q, n_{Q'}) = \delta_{n_Q,\overline{n_Q}}\delta_{n_{Q'},\overline{n_{Q'}}}$ gives

$$\sigma_{CA}^2 = \frac{1}{2}\sum_{Q=1}^{2P}\sum_{Q'=1}^{2P}\left[\overline{n_Q} - \overline{n_{Q'}}\right]^2 f_{Q'\overline{Q}} \tag{25}$$

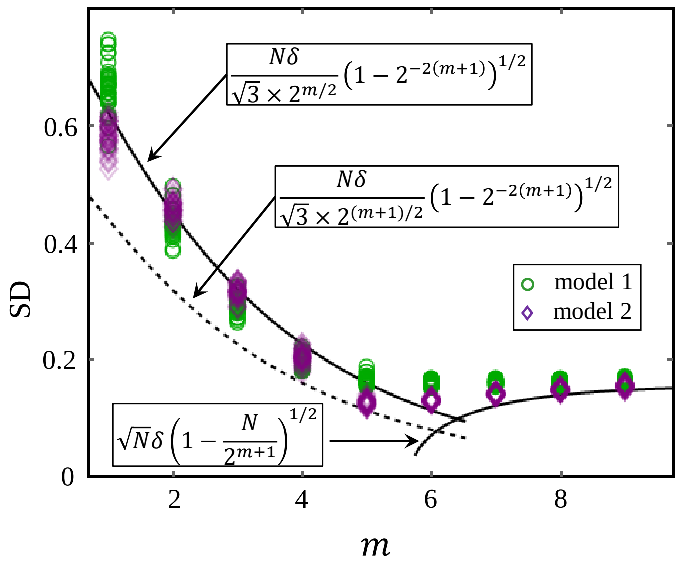

Fig. S8. Crowd-Anticrowd theory against numerical simulations for the model organism as a function of the agent capability m, for $N = 101$ agents, $s = 2$ and $\delta = \pi/2N$. The analytic forms of the fluctuations in the step-rotation are shown corresponding to $\sigma_{CA}^{\text{upperbound}}$ (upper solid curve), $\sigma_{CA}^{\text{lowerbound}}$ (lower dashed curve) and $\sigma_{CA}^{\text{flat } f,\text{ high } m}$ (solid curve for $m > 5$). All of them multiplied by the constant factor δ. The numerical simulations were obtained from different simulation runs form model 1 (green circles) and model 2 (purple diamonds).

where the function $f_{Q',\overline{Q}}$, which is the probability that the strategy with label Q' is anticorrelated to strategy Q, still needs to be specified. Now we assume that the resulting disorder in the popularity ranking (as compared to the original virtual-point ranking) is so strong, that the probability that Q' is anticorrelated to Q becomes *independent* of the label Q' and is hence given by $1/(2P)$. Hence the anticorrelated strategy to Q could lie *anywhere* in the strategy list $\{Q'\} = 1, \ldots 2P$. In this sense, this is the opposite limit to the upper-bounded case corresponding to the flat disorder matrix at small m. Hence instead of being a delta-function at $Q' = 2P + 1 - Q$, it now follows that $f_{Q',\overline{Q}}$ has a flat form given by $1/(2P)$. In this limiting case we have

$$\sigma_{CA}^{\text{lowerbound}} = \frac{N}{\sqrt{3}\times 2^{(m+1)/2}}(1-2^{-2(m+1)})^{\frac{1}{2}} \tag{26}$$

III. Non-flat quenched disorder matrix Ψ, large m

For larger m, the standard deviation in the number of agents in a given bin is now similar to the mean value. (By large m it is meant that the number of strategies is greater than $N.s$, i.e. $2.2^m > N.s$). Furthermore, there tend to be either 0 or 1 agents in each box (Q, Q'). In this limit, there will tend to be $O(N)$ crowds, with each crowd having $O(1)$ agent. Hence the popularity ordering is highly degenerate since $n_Q = 0, 1$ for all Q. Since the anticorrelated strategy to Q could be anywhere, we have $f_{Q',\overline{Q}} = 1/(2P)$. Using Equation 25 then gives

$$\begin{aligned}\sigma_{CA}^2 &= \frac{1}{2}\sum_{Q=1}^{2P}\sum_{Q'=1}^{2P}\left[\overline{n_Q}-\overline{n_{Q'}}\right]^2 f_{Q',\overline{Q}} \qquad (27)\\ &= \sum_{Q=1}^{N}\left[(\overline{n_Q}=1)-(\overline{n_{Q'}}=1)\right]^2\frac{N}{2P}+\left[(\overline{n_Q}=1)-(\overline{n_{Q'}}=0)\right]^2\frac{2P-N}{2P}\end{aligned}$$

where the sum is now performed over the N strategies with one agent subscribed. Simplifying this

expression gives

$$\begin{aligned}
\sigma_{CA}^{\text{flat } f,\text{ high } m} &= \left(\sum_{Q=1}^{N} \left[(\overline{n_Q} = 1) - (\overline{n_{Q'}} = 0) \right]^2 \frac{2P - N}{2P} \right)^{\frac{1}{2}} \\
&= \left(N. \frac{2P - N}{2P} \right)^{\frac{1}{2}} \\
&= \sqrt{N} \left(1 - \frac{N}{2^{m+1}} \right)^{\frac{1}{2}} \qquad (28)
\end{aligned}$$

where $P \equiv 2^m$ has been used. Figure S8 shows how the theory of fluctuations compare with numerical simulations of the step-rotation for different values of m. Finally, to get the estimate of the optimum capability m_o we look into the point where $\sigma_{CA}^{\text{flat } f,\text{ high } m}$ intersects with, for example, $\sigma_{CA}^{\text{lowerbound}}$. The result is presented in equation (3) in the main paper.
